# High Genetic Diversity Detected in Olives beyond the Boundaries of the Mediterranean Sea

**DOI:** 10.1371/journal.pone.0093146

**Published:** 2014-04-07

**Authors:** Mehdi Hosseini-Mazinani, Roberto Mariotti, Bahareh Torkzaban, Massoma Sheikh-Hassani, Saeedeh Ataei, Nicolò G. M. Cultrera, Saverio Pandolfi, Luciana Baldoni

**Affiliations:** 1 National Institute of Genetic Engineering and Biotechnology, Tehran, Iran; 2 CNR - Institute of Biosciences and Bioresources, Perugia, Italy; National Institute of Plant Genome Research, India

## Abstract

**Background:**

Olive trees (*Olea europaea* subsp. *europaea* var. *europaea*) naturally grow in areas spanning the Mediterranean basin and towards the East, including the Middle East. In the Iranian plateau, the presence of olives has been documented since very ancient times, though the early history of the crop in this area is shrouded in uncertainty.

**Methods:**

The varieties presently cultivated in Iran and trees of an unknown cultivation status, surviving under extreme climate and soil conditions, were sampled from different provinces and compared with a set of Mediterranean cultivars. All samples were analyzed using SSR and chloroplast markers to establish the relationships between Iranian olives and Mediterranean varieties, to shed light on the origins of Iranian olives and to verify their contribution to the development of the current global olive variation.

**Results:**

Iranian cultivars and ecotypes, when analyzed using SSR markers, clustered separately from Mediterranean cultivars and showed a high number of private alleles, on the contrary, they shared the same single chlorotype with the most widespread varieties cultivated in the Mediterranean.

**Conclusion:**

We hypothesized that Iranian and Mediterranean olive trees may have had a common origin from a unique center in the Near East region, possibly including the western Iranian area. The present pattern of variation may have derived from different environmental conditions, distinct levels and selection criteria, and divergent breeding opportunities found by Mediterranean and Iranian olives.These unexpected findings emphasize the importance of studying the Iranian olive germplasm as a promising but endangered source of variation.

## Introduction

The olive (*Olea europaea* L. subsp. *europaea* var. *europaea*) is known as a symbol of the Mediterranean basin, but it also grows towards the east, to western Asia, and on to Georgia, Azerbaijan and Iran, which is known as one of the most eastern olive-producing countries. There is a long history of olive cultivation documentation in the Middle East, including citations in religious texts and descriptions by archaeo-botanists [1]. Recent biogeographical and archaeological studies have limited the area of origin for olive tree domestication to the western Mediterranean basin during the late Neolithic and Chalcolithic period [2], [3]. Other works have placed it in the Near East [4], [5], [6], although this area was broader in the past and spanned the southern Caucasus to the Iranian plateau [7].

Olive cultivation developed significantly approximately 5,000 ya (years ago) in the eastern Mediterranean, spreading either to the island of Cyprus and towards Anatolia or from the island of Crete towards Egypt. Olives subsequently expanded into the western Mediterranean and were conveyed by Phoenicians, Greeks and Romans [8], [9]. A recent study based on chloroplast markers [6] demonstrated that as many as 90% of present-day cultivars are characterized by the same haplotypes, suggesting a human-mediated dispersal of this chlorotype from the eastern Mediterranean into western localities. In addition, archaeological and historical evidences support the spread of cultivated olive trees from the Near East to the western Mediterranean [1], [10].

Conversely, the early history of olive trees in Iran is shrouded in uncertainty. Pollen remains demonstrated the presence of olives in the western area of Iran starting approximately 4,300 ya, coinciding with the onset of the Bronze Age civilization, when olive pollen is appeared simultaneously with other tree crop species, such as *Pistacia*, *Juglans* and *Platanus* [11]. This evidence may support the idea that the first domesticated olive was more likely to have spread with other crops, first to the whole Levant [12] before being progressively disseminated to the western Mediterranean.

During the 10th and 11th centuries, olives were cultivated in the Iranian areas of Nisapur, Gorgan, Deylam, Ramhormuz, Arrajan, and Fars. This distribution likely reflects the agricultural situation as it existed in pre-Islamic Persia [13]. Olives were later most likely neglected throughout the Iranian plateau for a very long time for various historic and economic reasons. In the 19th century, olive cultivation in Iran was mainly concentrated along the Caspian provinces, and during the first half of the 20th century, olives were grown solely in the Manjil, Rudbar and Tarom districts of the Elburz Mountains. At present, the olive crop area in Iran covers approximately 110,000 hectares, and it is still established along the Caspian Sea and northern provinces from Zanjan to Golestan. Thus, the country has a relatively small olive growing area [14]; furthermore, a massive introduction of allochthonous varieties has recently been reported [15].

Discrimination of olive genotypes is widely performed by the use of SSR markers [16], and numerous Iranian olive accessions have been analyzed in this way. In particular, SSR markers, used in a survey of 92 accessions of Iranian olive varieties, from the northern provinces of Gilan, Zanjan and Qazvin, uncovered the presence of cases of identity among genotypes carrying different names, as well as cases of variations within cultivars with the same name [17]. Another work, on different genotypes corresponding to the three most common names of Iranian cultivars (Golooleh, Shengeh and Rowghani), showed again high variability within accessions carrying the same name, underlining the discrimination power of SSR markers and the need to clarify the cases of homonymy [18]. It was also previously observed that a large set of Iranian cultivars grouped separately when compared with 6 [19] and 30 Mediterranean cultivars [20].

Samples belonging to O. *europaea* subsp. *cuspidata* occurred as spread plants in southern and south-eastern Iran. It was demonstrated that *cuspidata* samples from Kerman contain a chlorotype common to samples from China, Pakistan, Oman and Ethiopia [21], [22], whereas other *cuspidata* representatives collected from the Hormozgan province showed two different forms that were distinguished by morphological and RAPD marker data [23].

Apart from cultivated and *cuspidata* trees, many other olive plants of unknown origin exist in Iran, most of which are found in regions far from cultivated areas and are represented by a single or a few trees. They may have large trunks, medium or large fruits [24], and grow under extreme climatic conditions. Some of these plants occur in Zanjan and Kerman, two of the coldest provinces in the country, which have average minimum temperatures of −8°C and −7.1°C, respectively, as well as in Khuzestan and Hormozgan, where the maximum temperatures are registered at 42° and 46.5°C. It has been reported that more than 50% of the olive plants in Iran grow in areas with an annual rainfall lower than 300 mm. Because of these climatic constraints, the best olive growing conditions in some provinces are found at altitudes higher than 2,500 m above sea level (asl) (Mediterranean basin olive cultivation may be found at an altitude of 700 m asl at most).

These olives are spread from the north-western to the south-eastern provinces of Iran and are referred to as ecotypes in this text. A wide repertoire of these ecotypes has been analyzed, for the first time, in this study using a selected set of chloroplast and SSR markers, and these data were compared with a selection of the most important Iranian varieties and with Mediterranean cultivars that are considered particularly representative for their wide diffusion, genetic diversity and commercial importance [16]. The objectives of this work include the following: i) elucidating the origin of these genotypes, ii) determining the relationships between Iranian ecotypes and cultivated varieties, and iii) verifying their contributions to the development of the existing variability.

## Materials and Methods

### Plant Material

**Figure 1:**
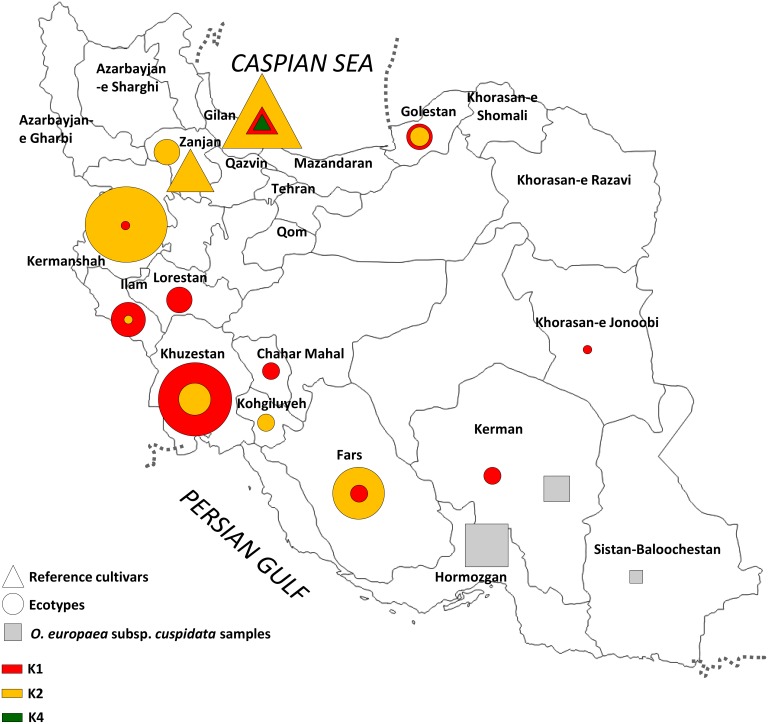
Map of Iran with the main provinces where the olive samples were collected. Triangles correspond to main cultivars; circles indicate ecotypes; grey squares represent O. *europaea* subsp. *cuspidata* samples. Different colors were related to the K groups derived from structure analysis, corresponding to the Iranian cultivars and ecotypes. Symbol size was related to the number of samples.

One hundred and five olive samples: 20 reference Iranian cultivars (Fishomi, Mari, Zard, three under Khorma and Rowghani names, five as Golooleh and six as Shengeh) and 85 trees collected from different geographical locations, were included in this analysis (Table 1). Reference cultivars were selected based on a previous characterization of Iranian olive cultivars [17], [25] and samples were derived from the Tarom and Rudbar collections. The 85 samples represent a wide range of olive trees from the Iranian plateau, directly collected from their original sites of spreading. Each ecotype sample represents a putative different genotype and was named according to the common name of the tree location (distinguishing each tree by different numbers). Although these trees do not cover the wide range of olive variability present in Iran, they certainly represent a broad repertoire of olives across the country, some of which under abandoned or wild or semi-natural growing conditions. A map indicating the provinces and sample collection sites is shown in Figure 1. Sampling of olive ecotypes has been performed according to the indications of the Iranian Ministry of Agriculture, the authority that has enabled their localization and collection. Iranian samples have been compared with 77 of the most important olive cultivars in ten Mediterranean countries, as previously selected and analyzed [16].

**Table 1:** List of the 105 Iranian olives analyzed, germplasm collections where reference cultivars were collected and corresponding accession numbers.

Reference cultivars	Collection	Accession number
FISHOMI	Tarom*	IRCul-03
GOLOOLEH-I	Rudbar**	IRCul-08
GOLOOLEH-II	Rudbar	IRCul-09
GOLOOLEH-III	Rudbar	IRCul-10
GOLOOLEH-V	Rudbar	IRCul-11
GOLOOLEH-VIII	Rudbar	IRCul-12
KHORMA-I	Rudbar	IRCul-19
KHORMA-II	Rudbar	IRCul-20
KHORMA-IV	Rudbar	IRCul-21
MARI	Tarom	IRCul-02
ROWGHANI-I	Tarom	IRCul-04
ROWGHANI-III	Tarom	IRCul-05
ROWGHANI-V	Tarom	IRCul-07
SHENGEH-I	Rudbar	IRCul-13
SHENGEH-II	Rudbar	IRCul-14
SHENGEH-III	Rudbar	IRCul-15
SHENGEH-IV	Rudbar	IRCul-16
SHENGEH-V	Rudbar	IRCul-17
SHENGEH-VI	Rudbar	IRCul-18
ZARD	Tarom	IRCul-01

Ecotypes	Sites of collection (province)	
Koohlak	Chahar-Mahal	
Ourmand	Chahar-Mahal	
Soonak	Chahar-Mahal	
Dehghan	Fars	
Derak	Fars	
Dezful-Shiraz	Fars	
Kazerooni	Fars	
Shapoor-I	Fars	
Shapoor-II	Fars	
Shiraz	Fars	
Tokhm-Kabki	Fars	
Varak	Fars	
Alazman	Golestan	
Ghazanghayeh	Golestan	
Kalaleh	Golestan	
Lamesk	Golestan	
Tooskestan	Golestan	
Kolah Faraj-I	Ilam	
Kolah Faraj-II	Ilam	
Malekshahi	Ilam	
Nargesi-I	Ilam	
Nargesi-II	Ilam	
Siab darvish	Ilam	
Charfarsakh-I	Kerman	
Charfarsakh-II	Kerman	
Shahdad	Kerman	
Banavare-2	Kermanshah	
Banavare-3	Kermanshah	
Banavare-5	Kermanshah	
Banavare-6	Kermanshah	
Banavare-7	Kermanshah	
Dalahoo-1	Kermanshah	
Dasht-e-Deireh-1	Kermanshah	
Dasht-e-Deireh-2	Kermanshah	
Deh Sefid-6	Kermanshah	
Deh Sefid-8	Kermanshah	
Gilan-e-Gharb-1	Kermanshah	
Park-e-Sarpol-1	Kermanshah	
Park-e-Sarpol-8	Kermanshah	
Rijab-1	Kermanshah	
Fosoon	Khorasan-e-Jonoobi	
Aghili	Khuzestan	
Avend	Khuzestan	
Bard-I	Khuzestan	
Bard-II	Khuzestan	
Bard-III	Khuzestan	
Bard-IV	Khuzestan	
Bard-V	Khuzestan	
Chelisad	Khuzestan	
Dakal-281	Khuzestan	
Dezful-292	Khuzestan	
Dezful-Safiabad	Khuzestan	
Ketfe-e-Gooshe	Khuzestan	
Maryab	Khuzestan	
Mavi-I	Khuzestan	
Mavi-II	Khuzestan	
Qiup	Khuzestan	
Deli-ba-Yar-3	Kohgiluyeh & Boyerahmad	
Tang-e-Tamoradi-6	Kohgiluyeh & Boyerahmad	
Deh Sefid-6	Lorestan	
Deh Sefid-8	Lorestan	
Sepid-Dasht-1	Lorestan	
Sepid-Dasht-2	Lorestan	
Sepid-Dasht-3	Lorestan	
Dastjerd-2	Zanjan	
Dastjerd-3	Zanjan	
Dastjerd-4	Zanjan	
RG-2	Zanjan	

*O. europaea* subsp. *cuspidata*	Sites of collection (province)	
*Aghin*	Kerman	
*Torang-121*	Kerman	
*Torang-122*	Kerman	
*Torang-123*	Kerman	
*Torang-128*	Kerman	
*Torang-IV*	Kerman	
*Bokhoon-1*	Hormozgan	
*Bokhoon-2*	Hormozgan	
*Bokhoon-3*	Hormozgan	
*Bokhoon-4*	Hormozgan	
*Bokhoon-5*	Hormozgan	
*Bokhoon-6*	Hormozgan	
*Bokhoon-7*	Hormozgan	
*Bokhoon-8*	Hormozgan	
*Bokhoon-9*	Hormozgan	
*Bokhoon-10*	Hormozgan	
*Beerk-I*	Sistan-Baloochestan	
*Beerk-II*	Sistan-Baloochestan	
*Hooshak*	Sistan-Baloochestan	

Each ecotype sample has been distinguished by the name of collection site and by different numbers when more than one tree per site was sampled. Provinces where ecotypes and *Olea europaea* subsp. *cuspidata* samples occur were also reported.

*National Olive Collection of Tarom (Zanjan province).

**Olive Collection of Rudbar (Gilan province).

### Genotyping by SSR Markers

The total DNA was extracted from olive leaves using a GeneElute Plant Genomic DNA Miniprep Kit (SIGMA) according to the manufacturer's instructions. Samples were analyzed by selecting the best 11 ranked SSR loci [16], which represent (at present) the most informative SSRs for olive cultivar discrimination. This method is able to distinguish among more than 99% of analyzed varieties. PCR amplifications were performed in a reaction volume of 25 μl containing 25 ng of template DNA, 10× PCR buffer, 200 μM of each dNTP, 10 pmol of each primer (forward primer labeled with FAM, NED, PET and VIC fluorescent dyes) and 2 U of Perfect Taq DNA Polymerase (5 PRIME, eppendorf). Amplifications were performed with the PCR System 9600 (Applied Biosystems, Foster City, CA, USA) using the following cycling conditions: an initial denaturation step at 95°C for 5 min, followed by 35 cycles of 95°C for 30 sec, annealing temperatures (as suggested by the authors) for 30 sec and 72°C for 25 sec, followed by a final elongation step at 72°C for 30 min. The resulting PCR products were first visualized by 2% agarose gel electrophoresis and then loaded into an ABI 3130 Genetic Analyzer (Applied Biosystems, Foster City, CA, USA). Output data were analyzed using GeneMapper 3.7 (Applied Biosystems).

### Genotyping by Chloroplast Markers

For the chloroplast genotyping, all Iranian samples were analyzed by using the most polymorphic chloroplast markers, including twenty-four length markers (including 29 SSRs or indels) and twenty SNPs. Markers were selected according to previous published data [6], [26], [27], after eliminating linked or low informative polymorphisms for olive varieties. A new primer set has been developed to amplify and detect length and SNP chloroplast polymorphisms (Table S1).

To discriminate between different lengths, a fluorescent tail was annealed to each forward primer using two-step PCR as follows: first, 31 cycles of regular amplification were performed at 60°C Tm, followed by 14 tail annealing cycles at 52°C. Negative controls (no template DNA) were included in all experiments. All other conditions, which are not specified here, were taken from the SSR amplification protocol. For SNP identification, the SNaPshot Multiplex System technique was used according to the manufacturer’s instructions (Life Technologies). The first PCR was performed using the same amplification conditions as those used for the SSRs. After this step, pre-amplicons were purified to remove primers and unincorporated dNTPs using ExoSAPIT (GE* Healthcare ExoSAPIT* PCR Purification Kit), and the next cycle was performed at 37°C for 45 min with a final step at 75°C for 15 min.

The resulting profiles were compared with the main chlorotypes of cultivated Mediterranean olives [6], [26], [27].

Representative samples selected from some Asian *Olea europaea* subspecies *cuspidata* samples (CNR-IBBR collection) were also included in the comparison to confirm the presence of the *cuspidata* subspecies in this area, as previously reported [21], [22], [23]. This action allowed for a distinction between the chlorotypes of site-specific ecotypes and those of *cuspidata*. Because the plastidial analysis results confirmed the distinctions obtained by morphological evaluation, these samples are referred to as *cuspidata* samples (Table 1).

### Chloroplast and SSR Allele Sequencing

A subset of chloroplast amplicons representing the three different chlorotypes detected by fragment and SNP analysis was sequenced directly to verify the correspondence between the sequence and length of fragments.

Nuclear SSR fragments, which yielded lengths never before detected in Mediterranean varieties, were also sequenced to determine whether the new polymorphisms were a consequence of changes occurring in the repetitive motifs or along the flanking regions and to assign correct allele sizes. The SSR fragments of subsp. *cuspidata* samples were also included in the sequencing task to verify sequence correspondence and to detect possible polymorphisms in comparison with the subsp. *europaea* genotypes.

After recovering DNA from agarose band excision by agarose GelExtract mini kit 50 preps (5 Prime, Eppendorf) according to the manufacturer’s protocol, microsatellite amplicons were directly sequenced using the Big Dye Terminator technique (Applied Biosystems) on an ABI Prism 3130 Automatic Sequencer (Applied Biosystems, Foster City, CA, USA). Alleles differing by a few repeats were not easily distinguishable on agarose gels because of the reduced distance between the two alleles, and they were cloned into a pGEM-T Easy Vector System I (PROMEGA) and transformed into *E. coli* XL1 blue cells. PCR amplifications and cloning products (with at least 10 colonies/allele) were run on an ABI Prism 3130 Automatic Sequencer (Applied Biosystems, Foster City, CA, USA).

### Data Analysis

GenAlEx 6.5 [28] detected the total alleles for each locus and for each population (reference cultivars, ecotypes, Mediterranean varieties and *cuspidata* samples), the number of alleles (Na), number of effective alleles (Ne), Shannon’s information index (I), observed (Ho) and expected (He) heterozygosity, and fixation index (F).

The polymorphism information content (PIC) was calculated using Microsatellite 3.1.1 [29] to understand the ability of select SSRs to discriminate among the analyzed genotypes.

FreeNA [30] and Micro-Checker version 2.2.3 [31] were used to estimate the presence of null alleles at the eleven loci, and long allele drop out or scoring errors were caused by scattering in the SSR profiles, although such alleles were apparently responsible for heterozygote deficiencies.

An analysis of MOlecular VAriance and F-statistic values (Fis, Fit and Fst) was validated with a permutation test, performed using GenAlex. The same software and GENEPOP V. 4.2 [32], [33] were used for a Chi-Square test and Fisher’s method to confirm the Hardy Weinberg (HW) equilibrium results.

A pairwise population matrix for Nei’s genetic distance and identity (NeiP) and the matrix of fixation index values (FstP) were calculated using GenAlEx. Private alleles for each population were also detected, highlighting the difference within samples and among loci.

FreeNA was also used to estimate the Cavalli-Sforza and Edwards genetic distances [34] for each pair of populations by using and not using the ENA correction [30] and for assessing global and pairwise comparison populations by Fst. Fstat analyses were conducted to perform genetic difference, allelic richness, and Fst and Fis evaluations by locus and population.

The genetic distance matrix, performed using GenAlex, was used to construct a dendrogram in Darwin v.5 software [35] by using the weighted neighbor joining method with 10,000 bootstrap replications, including or excluding *cuspidata* samples to clarify the relationships between Iranian and Mediterranean genotypes of *Olea europaea* subspecies *europaea*.

To determine the parentage among genotypes with different genetic backgrounds and to establish parent-offspring relationships among all genotypes, a parentage analysis was conducted using CERVUS version 3.0.3 [36] by analyzing all combinations and accounting only for those offspring generated by direct crossing.

The SSR data of all samples have also been analyzed using the STRUCTURE 2.3.4 software [37] with 1,000 replicate MCMCs and a burn-in period of 5,000 iterations, followed by a sampling period of 10,000 iterations applied for each K. The program was run with K values ranging from 1 to 10, considering the maximum number of expected populations. Upon determination of the best K value for admixture analysis, a bar graph was performed.

To determine whether Iranian cultivars and ecotypes and Mediterranean cultivars have undergone a genetic drift, we used one-sided group comparisons in the FSTAT software [38] with 1,000 permutations to test for significant differences in allelic richness (AR) and gene diversity (He) between the two groups. The excessive heterozygosity relative to the allele numbers was expected after a bottleneck, and it was computed using BOTTLENECK version 1.2.02 [39], according to the Infinite Alleles Model (IAM), the Stepwise Mutation Model (SMM) and a two-phase model of mutation (TPM) with a 70% stepwise component.

## Results

### Chloroplast Variation Analysis

An analysis of chloroplast markers has allowed for the detection of three different chlorotypes among Iranian samples. After checking their correspondence with different previously published chlorotypes and with the subspecies included in the analysis, all ecotypes and reference cultivars showed the same chlorotype as the ‘Frantoio’ cultivar [26], which corresponds to E1.1, the most common chlorotype (90%) [27] among the Mediterranean cultivars.

Conversely, all Hormozgan samples, two of which were from Sistan-Baloochestan and five from Kerman, carried the same subsp. *cuspidata* chlorotype from Nepal, whereas one from Kerman and one from Sistan-Baloochestan exhibited the subsp. *cuspidata* chlorotype from India (Table S2). All of these provinces are adjacent and lay in the southeastern corner of the country bordering the Indian sub-continent.

A deep sequencing subset analysis of each haplotype group was performed on several chloroplast regions, confirming their perfect correspondence with the reference chlorotypes (data not shown). No other chlorotype forms noted in previous studies have been found in the Iranian samples.

### Frequency and Private Alleles

All 11 microsatellite markers used in this study displayed high polymorphism and discrimination powers, as demonstrated by the high PIC values for each SSR marker, which ranged from a low of 0.72 (DCA14) to a high of 0.93 (DCA09).

The allocation of all samples into four distinct populations, including reference cultivars, ecotypes and *cuspidata* samples from Iran as well as Mediterranean varieties (Table 2), resulted in an allelic number ranging from 7.27 for the reference cultivars to 12.45 for the Iranian ecotypes. The mean value for effective alleles across populations was almost half that of the total alleles (5.32) and ranged from 4.23 for the Iranian reference cultivars to 6.56 for the ecotypes.

**Table 2:** Number of total (Na) and effective (Ne) alleles, expected (He), observed (Ho) heterozygosity and Fixation index (F), for the SSR markers for each of the three Iranian groups and the Mediterranean olives.

		Iran			Mediterranean cultivars
	Mean	Reference cultivars	Ecotypes	*cuspidata*	
**Na**	10.09	7.27	12.46	8.36	12.27
**Ne**	5.32	4.23	6.56	4.66	5.84
**Ho**	0.72	0.72	0.72	0.59	0.85
**He**	0.76	0.73	0.79	0.71	0.81
**F**	0.05	0.01	0.07	0.17	−0.05

Considerable numbers of private alleles were detected in the GenAlex analyses. The Iranian and Mediterranean samples showed 218 SSR alleles, 83 of which were private to Iran and 36 to the Mediterranean genotypes (Table 3). Thirty-six were specific to the main cultivars and/or ecotypes, 16 were shared by the three Iranian populations and 31 were specific to *cuspidata* samples (Table S3). Among these 83 private alleles, which represented 44.86% of the total alleles in Iranian samples, 44 were within the expected, previously detected range in the Mediterranean cultivars and 39 were outside this range, considering those with a shorter (down to 30 bp) or longer (up to 40 bp) length than previously observed [16]. Some alleles turned out to be private to distinct Iranian populations; there were two private alleles for the reference cultivars, 21 for the ecotypes, and 13 that were present in both populations. When considering the main cultivars and ecotypes as a single group, these private alleles represent 19.46% of the total alleles in this group. Finally, 31 were exclusively detected within the *cuspidata* population, representing 34.83% of the total alleles in this group; nine were common to cultivars-or-ecotypes and *cuspidata*, and six were found in all Iranian samples (Table S4). The percentage of shared alleles between the Iranian cultivar-ecotypes and Mediterranean cultivars was 50.27%. From a direct comparison of Mediterranean varieties and *cuspidata* samples, only 24.73% were shared. The percentage of private alleles in each population or group of populations shows that the private alleles that are present within the ecotypes and reference Iranian varieties occurred in 14.58% of the total alleles of this group; the common alleles between the ecotypes and cultivars with *cuspidata* occurred in 7.19% of the total alleles. The occurrence of alleles shared between Iranian ecotypes and cultivars with Mediterranean varieties was 78.23%, and 57.51% of the alleles were shared between *cuspidata* and the Mediterranean cultivars.

**Table 3:** All SSR allele lengths.

Locus	Total alleles	Allele length (bp)
DCA3	26	**227–229–**232–**235–**237–239–**241–**243–245–**247–**249–**251–**253–255–**257–259–261–263–272–279–281–283–288–290–293–297**
DCA5	13	192–194–198–200–202–204–206–208–210–212–214–**218–220**
DCA9	27	162–166–**169–**172–**174–** 176–**178–**180–182–184–186–188–**190–**192–194–**196–**198–**200–**202–204–206–208–210–**212–**216–**218–220**
DCA14	16	**143–145–147–149–**173–175–**177–**179–181–183–185–187–189–191–193–197
DCA16	33	**122–**124–126–128–**130–133–135–137–139–142–144–** 146–**148–**150–154–156–158–**160–162–** 166–170–**172–**174–**176–** 182–**200–206–** 210–**216–218–220–222–226**
DCA18	18	159–163–**165–**167–169–171–173–175–177–179–181–183–185–187–**193–195–198–207**
EMO-90	11	**182–**184–186–188–190–**192–**194–**196–**198–**200–**202
GAPU71B	14	**119–**121–124–127–130–**132–**136–**138–140–** 142–144–**146–** 148–150
GAPU101	15	182–191–193–**195–**197–199–201–**203–205–**207–**209–217–**219–**221–229**
GAPU103A	21	136–139–141–**144–146–148–**150–**154–** 157–159–162–172–174–177–179–181–184–186–**188–**190–192
UDO-043	24	**164–168–** 170–172–174–176–178–180–184–186–188–196–198–200–202–204–206–208–210–212–214–216–218–**220**
Total	218	

83 alleles private (in- and out-of-range) to the Iranian samples are highlighted in bold.

Underlined numbers refer to 33 alleles private to the Mediterranean cultivars.

**Figure 2:**
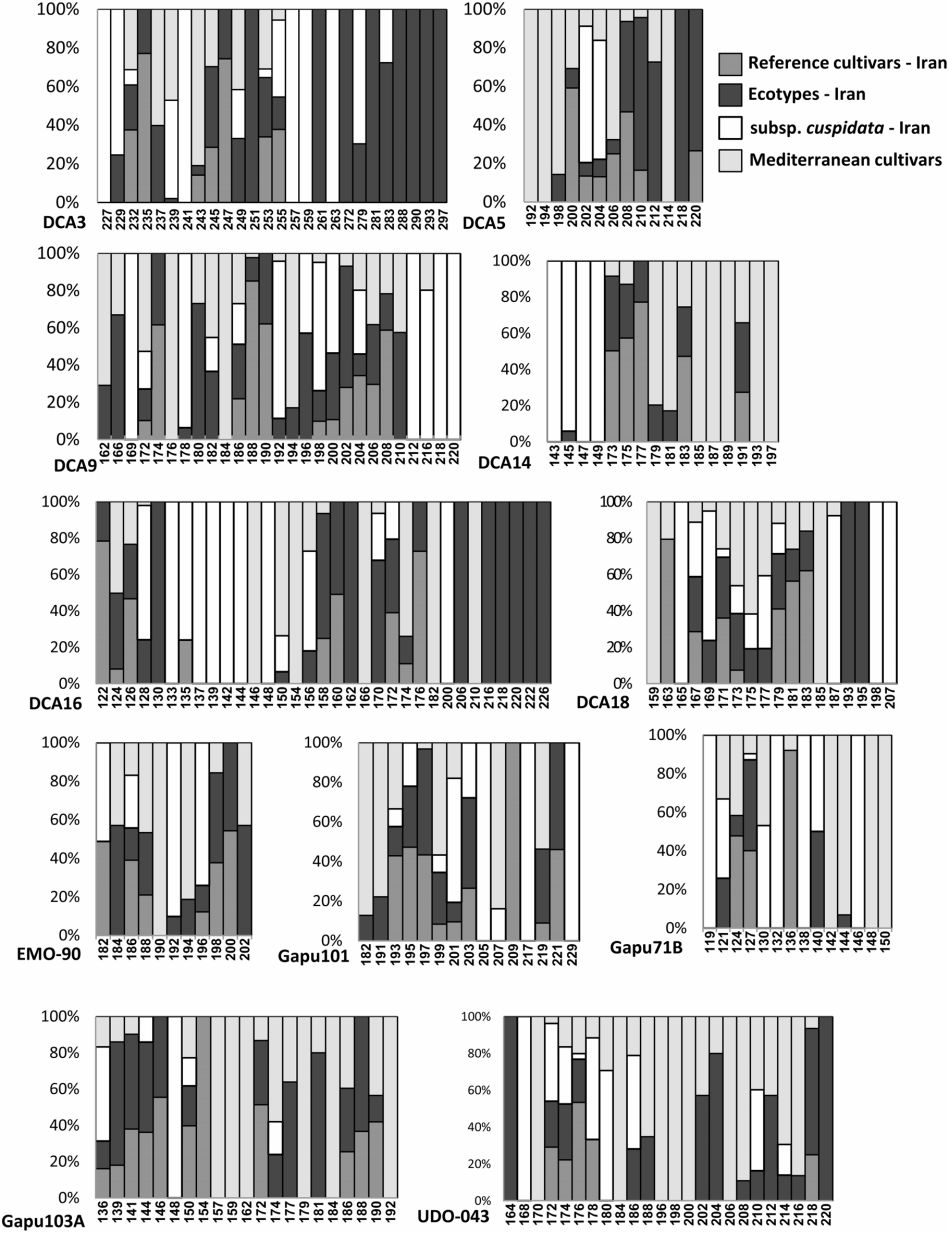
SSR allele distribution across populations at each locus. Numbers refer to the allele lengths.

Specifically, alleles private to the ecotypes were represented within loci DCA3, DCA5, DCA16, DCA18 and UDO-043, whereas only two alleles were private among the reference cultivars in GAPU101 and GAPU103A (Figure 2). Private alleles showed the highest frequency within the *cuspidata* samples, despite the small size of this population, and loci DCA9, DCA14 and GAPU71B showed alleles that were exclusively present in this group. Only two ecotypes, i.e., Varak from Fars and Ourmand from Chahar-Mahal, held the 145 alleles for DCA14, representing the typical length occurring solely in all *cuspidata* samples.

Sequencing some of the new alleles on a few ecotypes and *cuspidata* samples revealed changes in the motif repeats and/or the flanking regions (Table 4). In particular, a different number of repeats was observed only in DCA16, DCA18 and UDO-043, and the DCA14 alleles did not hold the TAA repeat, which is typical of this locus.

**Table 4:** List of sequenced private out-of-range SSR alleles, corresponding genotypes carrying the sequenced allele and accession numbers.

Locus	Allele	Genotype	Accession Number
DCA3	279	Torang-128 (*cuspidata*)	JX514168
	297	Deli-ba-Yar-3 (ecotype)	JX514169
DCA14	147	Bokhoon-4 (*cuspidata*)	JX514170
	143	Beerk-I (*cuspidata*)	JX514171
DCA16	220	Maryab (ecotype)	JX514172
DCA18	207	Bokhoon-5 (*cuspidata*)	JX514173
UDO-043	164	Park-e-Sarpol-8 (ecotype)	JX514174

The Fst for each pair of populations was estimated using the FreeNA program, both using and not using the ENA correction [30]. As expected, the results demonstrated that the *cuspidata* group carried the highest values when compared with the other samples, yielding 0.151, 0.166, 0.184 when compared with the Mediterranean cultivars, Iranian ecotypes and Iranian cultivars, respectively. Significantly high values were also observed between Mediterranean cultivars versus Iranian varieties (0.102) and Iranian ecotypes (0.083). The lowest difference was revealed by comparing Iranian cultivars and ecotypes (0.032) (Table 5).

**Table 5:** Pairwise population Fixation index values (Fst) by FreeNA software with ENA correction.

		Iranian			Mediterranean cultivars
		Main cultivars	Ecotypes	*cuspidata*	
	**Main cultivars**	0.000			
**Iranian**	**Ecotypes**	0.032	0.000		
	***cuspidata***	0.184	0.166	0.000	
**Mediterranean cultivars**		0.102	0.083	0.151	0.00

An AMOVA analysis revealed a high percentage of molecular variance within individuals (85%), with 4% among individuals and 11% among the four groups. The results from the F-statistic test within all analyzed samples showed high values for Fst (0.117) and Fit (0.159) and a positive value (0.046) for Fis.

GENEPOP and the GenAlex software were used to verify the Hardy Weinberg (HW) equilibrium. It was observed that Iranian ecotypes do not mate randomly, giving highly significant probabilities for at least 9 of the 11 SSRs, according to the Chi-Square and probability tests (p<0.001).

Considerable diversity was uncovered when Fst pairwise values were run for three groups without *cuspidata*, with the results from the Fstat software yielding the lowest value (0.031) among Iranian cultivars and ecotypes, which increased when comparing ecotypes and Mediterranean cv (0.082) with a maximum for Mediterranean and Iranian varieties (0.104). Fis values were calculated using the same software and resulted in positive results for the Iranian cultivars and ecotypes and negative values for the Mediterranean varieties.

The mean observed heterozygosity result was lower than expected in all Iranian samples compared to the Mediterranean cultivars. The highest difference in observed heterozygosity was noted in *cuspidata* olives.

The fixation index showed positive values for all Iranian groups and negative values for Mediterranean cultivars.

A FreeNA and Microchecker analysis revealed the high probability of null alleles presence in several SSRs when all of the samples were analyzed together. The same analysis was subsequently performed for each group of samples, showing that the *cuspidata* group had the highest values, especially for the DCA14, DCA18 and GAPU101 loci (the values were obtained using both the Microchecker and FreeNA software). The estimated null allele frequency employed the Expectation-Maximization (EM) algorithm in the FreeNA software for the remaining three groups (main cultivar, ecotypes and Mediterranean varieties). It showed low values for the null allele frequency (with moderate values ranging from 0.05 to 0.2 [30] but yielded the highest estimation for DCA16 (0.08), though the estimation appeared to be quite low (< = 0.05) for the other SSRs. For this reason, all eleven microsatellite markers were considered in the genotype analyses of the three groups.

### Parentage

The kinship analysis performed by Cervus on all SSR data, including those of *cuspidata* (Table S5), allowed for the identification of 29 possible parentage cases. The cross between reference cultivars Rowghani-I and Shengeh-III resulted in the offspring of an ecotype from Zanjan (Dastjerd-4) and the highest LOD scores (log of odds) for any potential parentage relationships (parent/parent pair), yielding a value greater than zero, which conferred statistical significance to the data.

Nevertheless, in most cases, the ecotype Dastjerd-4 was identified as a parent in combination with different reference cultivars. It seems to have contributed to generating five offspring among the best 14 cases. A cross between Dastjerd-4 and reference variety Fishomi may have generated Park-e-Sarpol 1, though this sample was found near the province of Kermanshah, which is hundreds of kilometers from at least one putative parent. In another case, a cross of Dastjerd-4 and Khorma-IV may have generated Rowghani-III. The cross of reference cultivars Rowghani-I and Khorma-II resulted in Dezful-Safiabad, an ecotype from Khuzestan.

A search for the uniparental origin of genotypes, which was indicated in three reference cultivars (Fishomi, Golooleh-I and Rowghani-I) as the best direct parent candidates of several varieties, was included in this set of samples. Fishomi presented positive LOD scores for cultivars Shengeh-III, Khorma-II and Rowghani-III, followed by Rowghani-I and Khorma-IV, with two mismatches on 10 loci. The Fishomi cultivar emerged as a putative parent for some ecotypes, such as two Kermanshah samples (Park-e-Sarpol-1 and Gilan-e-Gharb-1), Dezful-Safiabad from Khuzestan and Dastjerd-4 from Zanjan. The two most widely cultivated varieties are grown in Iran for oil production, namely Rowghani-I and Golooleh-I. They yielded very high LOD scores and 1 mismatch on ten loci, indicating their likelihood of serving as reciprocal parents. Rowghani-I may also have generated Khorma-II, Golooleh-II, Rowghani-III and Shengeh-III. Golooleh-I seems to have contributed to the generation of ecotype groups Banavare-3, 5 and 6 from Kermanshah and Dastjerd-4 from Zanjan. Ecotypes Banavare-7 from Kermanshah and Dastjerd-4 may have also been derived from Rowghani-I, as observed in the bi-parental analysis.

### Genetic Relationships among Iranian Samples and Differentiating between Iranian and Mediterranean Genotypes

**Figure 3:**
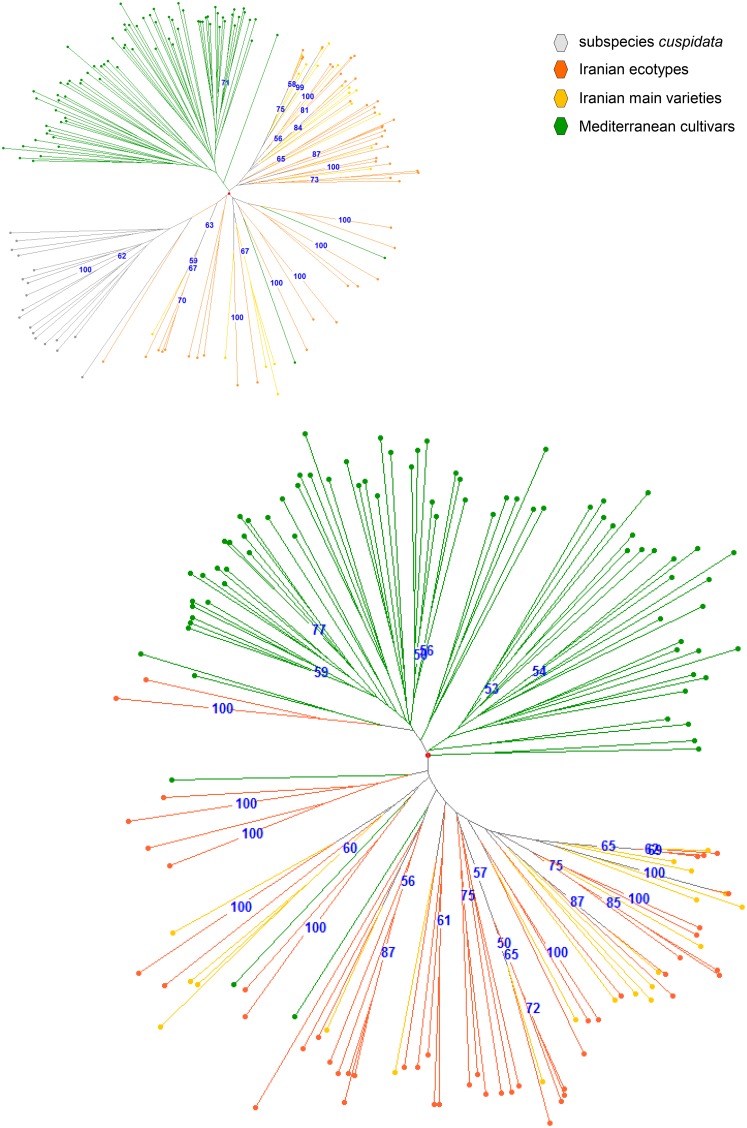
A DARWIN software elaboration performed using the Neighbor Joining method, with a bootstrap displayed threshold up to 60 (black numbers). a) Radial dendrogram visualization of the genetic relationships among reference cultivars (yellow), ecotypes (orange) and *cuspidata* (grey) samples of Iran and Mediterranean cultivars (green) derived from the SSR markers. b) Radial dendrogram without *cuspidata* samples, with each group maintained in the same color.

Two dendrograms were constructed using the Neighbor Joining (NJ) method with and without the *cuspidata* group (Figure 3). Both dendrograms showed a complete separation between Mediterranean cultivars and Iranian samples. Some exceptions were observed. Two Mediterranean cultivars clustered within the main group of Iranian varieties and ecotypes. Yun Celebi, from Turkey’s Anatolian region, is related to ecotypes from the Khuzestan and Kerman provinces. A second variety called Caiazzana from the Campania region of Italy appeared only in the setup without *cuspidata,* clustering with the group containing the Iranian cultivar Mari. In the Neighbor joining analysis without *cuspidata* samples, some ecotypes from Ilam province and a Ghazanghayeh sample from Golestan province clustered with two Greek cultivars (Kalamon and Konservolia).

The Iranian cultivar and ecotype results in both dendrograms were mixed, and the Varak ecotype from Fars was the only sample clustering within the *cuspidata* group when subsp. *cuspidata* samples were included, though it carries the typical chlorotype E1.1 from all Iranian cultivars and ecotypes.

The SSR profiles of some ecotypes were perfectly identical with each other, revealing the clonal origin of these trees, as confirmed in previous studies and by the high PIC values of all SSRs for the genotypes of interest. Most instances of identical profiles were observed among genotypes from a single province, with the first group from Khuzestan formed by the Bard and Mavi-II ecotypes and the second group containing Avend, Ketfe-e-Gooshe and Maryab. Another group was formed by Sepid-dasht-2 and 3 from Lorestan, and another contained by Kolahfaraj-I and II, Malek Shahi and Nargesi-I and II from Ilam. Two pairs of samples were formed by Rijab-1 and Gilan-e-Gharb-1 and by Deh-Sefid-6 and 8 from Kermanshah. Only one case of identity was observed between genotypes from different provinces, namely Shahdad and Charfarsakh-I and II from Kerman and Fosoon from Khorasan-e-Jonoobi. Additionally, two *cuspidata* plants from the Hormozgan province called Bokhoon-8 and 9 were identical to each other.

**Figure 4:**
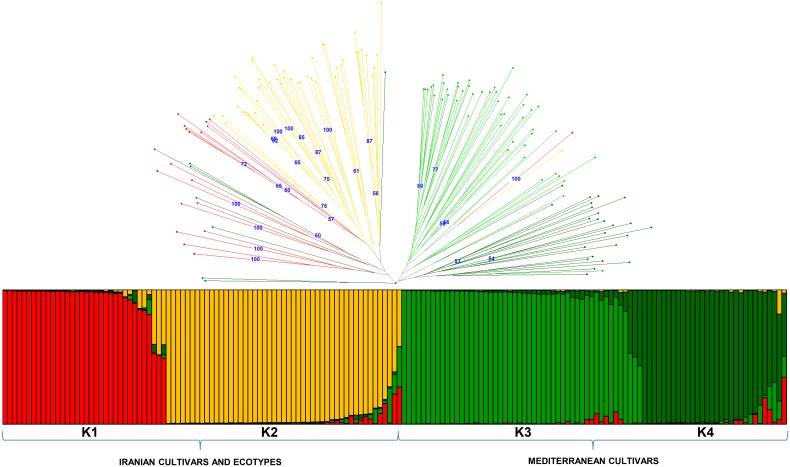
Bayesian clustering analysis of Iranian olive samples and Mediterranean cultivars, as performed using STRUCTURE. Bar plots correspond to the membership of four distinct clusters (K = 4), and the reported colors were chosen to better illustrate the difference between the Iranian and Mediterranean samples. Dendrogram reporting the same color as the four individuated K was also reported.

A Bayesian analysis by Structure (Figure 4) was performed without including *cuspidata* samples to eliminate the possibility of having an unrealistic output resulting from null alleles. Based on the K confidence, it was possible to distinguish the four most probable and distinct populations, where it was clear that Mediterranean and Iranian samples clustered completely separated and each pool included two distinct populations. Both Iranian populations included ecotypes and reference cultivars without a clear separation. Most genotypes were completely assigned to a single population, with a few exceptions. Only the two Mediterranean cultivars Yun Celebi and Caiazzana were intermixed with the Iranian genotypes, according to the Darwin dendrograms. The relationship between population structure output and NJ analysis was also graphically displayed (Figure 4) and the colors of the 4 groups found after Bayesian analysis have been maintained for all samples in the dendrogram, to give an indication of their correspondence. It was possible to observe an interesting clustering in some samples from different provinces such as Kermanshah and Zanjan, which included several main Iranian varieties. Samples from Khuzestan seemed to be separate from the others and to have an interesting relationship between western Ilam and north-eastern Golestan that was often close or intermixed. It the map of the country (Figure 1), the same membership colors of structure analysis were reported. It was observed an interesting geographical correlation for the ecotypes collected in north-western part of the country and the reference Iranian cultivars belonging to a single group (K-2).

According to the results obtained through a Bayesian population structure analysis, we analyzed the data with FSTAT by considering Iranian varieties and ecotypes to be a single population and Mediterranean cultivars to be another population. With FSTAT data providing an elevated value of allelic richness and based on a minimum sample size of 19 genotypes, the Iranian ecotypes were shown to have an average value of 9.48, which was higher than the of the Mediterranean varieties (8.28). The FIS values were positive in the Iranian ecotypes at most of the loci (9/11) and negative within the Mediterranean group (8/11 loci), with average values of 0.089 and −0.047, respectively (Table 6).

**Table 6:** Results of the FSTAT and Bottleneck analyses.

Population	Locus	A_R_	F_IS_	*H*_E_	*H*_Eq_	SD	DH/sd	*P*
**Iranian varieties and ecotypes**	DCA3	19,76	0,03	0.893	0.925	0.019	−1.755	0.021*
	DCA5	9,00	0,15	0.692	0.819	0.04	−3.16	0.012*
	DCA9	20,00	0,15	0.922	0.927	0.012	−0.408	0.301
	DCA14	8,00	0,13	0.705	0.793	0.044	−2	0.049*
	DCA16	19,74	0,29	0.873	0.926	0.018	−2.965	0.002*
	DCA18	11,91	0,18	0.786	0.869	0.023	−3.585	0.005*
	EMO-90	9,88	−0,07	0.801	0.839	0.035	−1.067	0.134
	GAPU71B	5,97	−0,10	0.422	0.723	0.068	−4.457	0.005*
	GAPU101	9,97	0,17	0.865	0.838	0.036	0.765	0.220
	GAPU103A	14,00	0,14	0.858	0.888	0.021	−1.478	0.082
	UDO-043	15,91	0,05	0.803	0.904	0.017	−5.992	0.000*
**Mediterranean cultivars**	DCA3	7,98	−0,10	0.842	0.792	0.048	1.05	0.107
	DCA5	10,57	−0,19	0.731	0.854	0.032	−3.88	0.009*
	DCA9	17,08	0,00	0.88	0.914	0.022	−1.508	0.031*
	DCA14	10,57	0,08	0.688	0.852	0.032	−5.082	0.002*
	DCA16	12,12	−0,01	0.85	0.878	0.024	−1.196	0.110
	DCA18	12,41	−0,04	0.861	0.877	0.024	−0.686	0.203
	EMO-90	6,71	−0,04	0.688	0.761	0.057	−1.265	0.104
	GAPU71B	8,55	−0,12	0.81	0.817	0.041	−0.167	0.346
	GAPU101	7,98	−0,15	0.851	0.791	0.049	1.219	0.037*
	GAPU103A	15,38	0,01	0.853	0.903	0.017	−2.907	0.017*
	UDO-043	19,80	0,04	0.89	0.928	0.015	−2.509	0.013*

Bottleneck analysis was performed with the heterozygosity excess model in the Mediterranean cultivars and the Iranian cultivars and ecotypes, according to the stepwise mutation model (SMM).

A_R_: allelic richness based on minimum sample size of 66 diploid individuals; F_IS_: Wright’s inbreeding coefficient. Indicative adjusted nominal level (5%) for P-value for Fis is 0.002. A_R_ and F_IS_ have been calculated through FSTAT.

H_E_: expected heterozygosity; H_Eq_: expected heterozygosity at mutation-drift equilibrium; SD: standard deviation of the mutation-drift equilibrium distribution of the heterozygosity; H_E_-H_Eq_/sd standardized difference for each locus. *P = 0.05, probability of deficit for the expected heterozygosity (HE). DH/sd has been calculated with Bottleneck.

A bottleneck analysis was performed by considering the Iranian ecotypes and cultivars to be two populations, which yielded negative DH/sd values in the group of ecotypes at 8 loci after SMM (P<0.05), with a heterozygosity deficiency confirmed by a Wilcoxon test (P<0.01). No significant values were obtained for the IAM in both groups or for the SMM in the Iranian cultivars.

## Discussion

The main goals of this investigation were to understand the relationships between Iranian olives and Mediterranean varieties, to shed some light on the origin of Iranian olives and to verify their contribution to the development of the current global olive variability.

Interest in this study was derived not only from the fact that olive cultivation in Iran is most likely as longstanding as it is in the Mediterranean [13], but it also arose because these trees can survive under extreme climate and soil conditions, lying scattered in the midst of arid lands and with extremely low or high temperatures, low water availability and altitudes reaching 2,500 m asl and the samples analyzed showed unexpected large-sized fruits, even bigger than those of best Mediterranean table-olive varieties, ruling out the possibility they belong to the typical wild form (*Olea europaea* subsp. *europaea* var. *sylvestris*) of the Mediterranean.

One hundred five Iranian samples, including primary varieties, ecotypes and *cuspidata* plants, were collected from different provinces. The samples were analyzed using the best set of SSR nuclear and the most discriminant chloroplast markers and were compared with a representative pool of Mediterranean olive cultivars.

### Relationships between Iranian and Mediterranean Cultivated Olives

The results from the population structure and Neighbor Joining analyses showed a distinct separation of all samples into two main clusters: one including all Iranian ecotypes and cultivars, and the other formed by the Mediterranean cultivars. Only a few exceptions to this general pattern were observed among the distinctive clusters, in which three Mediterranean cultivars were placed within the Iranian ecotype-cultivar branch. Regarding the few Iranian genotypes, clustering with Greek cultivars Kalamon and Konservolia, it is also worth noting the correspondence between the peculiar teardrop shape of Ghazanghayeh fruits [24] with those of the Greek cv. Kalamon, even if this relationship was not confirmed by Bayesian analysis.

SSR differences were mainly represented by a consistent set of private alleles detected within Iranian cultivars and ecotypes in most loci, and a few other private alleles were shared with the *cuspidata* samples.

The lack of excessive heterozygosity in the Mediterranean cultivars and Iranian samples, in which cultivars and ecotypes were considered together, gave no evidence of recent population bottlenecks in both populations under the stepwise mutation model. The positive fixation index and inbreeding coefficient values of the Iranian genotypes positioned the Iranian ecotypes far from Hardy-Weinberg equilibrium, suggesting that the olive trees Iranians have not had opportunities to hybridize with any other form, with the exception of the few plants of *cuspidata*, which may have contributed some alleles acting as a pollinator.

To the differences between the two groups observed with the nuclear SSR markers did not correspond an equal level of differentiation at plastid level. All Iranian cultivars and ecotypes, in fact, have shown the most common E1.1 chlorotype found within Mediterranean cultivars [6], [26], [27]. This introduction should have occurred only through seeds, allowing the conservation of the common chlorotype.

This structure of variation is in agreement with the statement that the Mediterranean was not a major primary center for olive tree domestication but, as highlighted by numerous works [2], [3], [4], [5], [6], the common center of origin should be confirmed in the Near East [1]. From this primary domestication center, cultivated olives may have spread in two opposite directions: towards the west, along the shores of the Mediterranean, or to the east, up to the Iranian plateau.

The maintenance of common alleles between Mediterranean and Iranian olives may have been possible for a long-living perennial crop as olive, exhibiting a significantly lower number of sexual generations than do annual seed crops occurring within a given period of time [40].

The analyzed samples may have undergone different routes of variation. In the Mediterranean, numerous cultivars may have been generated through crossings with local wild genotypes (*Olea europaea* subsp. *europaea*, var. *sylvestris*), representing Mediterranean refuge zones, and with other subspecies (such as *cerasiformis*, *guanchica*, *laperrinei* and *maroccana*) [6], [9], thus increasing the SSR allelic richness and chlorotype patterns.

In Iran, the high SSR variability may be explained by the conservation of the original gene pool as a consequence of a lack of selection, the application of selection criteria different from those applied in the Mediterranean, or the fixation of alleles linked to adaptations to extreme environmental conditions, while the presence of only one chlorotype can only be explained by the lack, in this area, of forms with different chlorotypes able to cross with local olives.

A portion of this variation may have been kept from *cuspidata* [41] or from local germplasm of still unknown olive refuge areas. All of these possible events may have increased the allelic richness in Iran, but the absence of gene flow, as in the Mediterranean basin, may have resulted in a high inbreeding coefficient in Iranian olives.

The geographic distance and different historical events of the two areas may have contributed to the maintenance of the separation between the Mediterranean and Iranian germplasm [42].

We can conclude that the founder effect is the most likely explanation for the genetic variability of the Iranian ecotypes.

### The Iranian *Olea Europaea* Subspecies *Cuspidata*

Based on the chloroplast data, all samples collected from south-eastern part of the country belonged to *O. europaea* subsp. *cuspidata*. The presence of *cuspidata* olives in these Iranian provinces has been previously reported by other authors [43], [44] and *cuspidata* samples from the Kerman province have been included in phylogenetic studies of the *Olea* complex [21], [22].

The chlorotype of most of these *cuspidata* olives corresponded to a Nepalese *cuspidata* sample, whereas the Aghin (Kerman) and Hooshak (Sistan-Baloochestan) samples belonged to the Indian *cuspidata*. The coexistence of different *cuspidata* chlorotypes may be the consequence of the spread of these forms from eastern countries such as India, Nepal or China.

Two *cuspidata* plants from the Hormozgan province were identical to each other, raising the possibility that they originated as natural re-sprouts of clonal shoots, as has occurred for other wild-growing plants of the subspecies *laperrinei* [45].

Some SSR alleles were shared among *cuspidata* samples and ecotypes and/or main varieties. In particular, a few ecotypes from Fars and Chahar-Mahal showed alleles characterizing all *cuspidata* plants, suggesting that hybridization between the two forms *europaea*×*cuspidata* may have been possible and may have naturally occurred in places where these forms may have cohabited for a long time. Further evidences of spontaneous hybridization occurring between cultivated plants and local *cuspidata* plants have been previously reported in South Africa [41]. It may be speculated that the barrier between the two subspecies is not complete and that some Iranian ecotypes may have been derived from a gene flow from *cuspidata* to local forms of *europaea*. The absence of *cuspidata* chlorotypes within ecotypes and varieties may be caused by the exclusive paternal contribution of *cuspidata* as a pollen donor. The importance of this issue requires further specific studies.

### Patterns of Diversity among Iranian Genotypes

A Bayesian population structure analysis showed a clear separation between Mediterranean and intermixed Iranian samples, represented by ecotypes and varieties. Bayesian analysis confirmed strong admixture values for some samples that were not assignable to any group, i.e., Varak, Caiazzana and Yun Celebi. The close relationships between ecotypes and reference varieties found with NJ analysis were partially confirmed by Structure results, where, considering the two Iranian sample groups, the second contained the greatest number of reference cultivars and almost all ecotypes from north-west provinces of the country, suggesting that the reference varieties most likely represent the most outstanding ecotypes selected by growers in the recent past.

Several cases of miscalling within the main cultivars, previously observed [17], [19], [20], have been confirmed by our analyses with very high PIC values, able to clearly distinguish the different genotypes.

The existence of identical genotypes occurring in provinces that were part of the Iranian Fertile Crescent, such as Ilam, Khuzestan, Kohgiluyeh and Fars, leaves open the possibility they represent remains of ancient olive orchards, also considering that olive cultivation was described as a central component of the wheat-based system [46], [47]. Furthermore, these clones were represented by ancient trees distributed in restricted areas (from 10 m to 40 Km), sometimes at regular distances or arranged in rows, as in the case of the Kolah Faraj samples from Ilam, corroborating the hypothesis they may represent escapees from cultivated plants. Conversely, other clonal ecotypes found at distant locations, especially in regions where olive cultivation is not presently reported neither evidences exist documenting a cultivation in the past, such as Kerman and Khorasan-e-Jonoobi, may be the result of vegetative propagation meant for ritual purposes in which olive was considered as a holy tree [48], allowing their survival until now, independently from their agronomic value.

Kinship parentage analysis reinforced the hypothesis of cultivation origin for most ecotypes, demonstrating a strong relationship among some main varieties, such as Golooleh, Fishomi, Rowghani, Khorma and Shengeh, showing that some ecotypes, such as Dastjerd 4 from Zanjan province, may represent direct progenitors of these cultivars.

A further confirmation derived from the evidence that ecotypes growing under very harsh environmental constraints, such as high and low temperatures (as in Khuzestan and Kerman, respectively) and low rainfall (Kerman), clustered jointly despite the great distances between the collection sites.

## Conclusions

An unexpected level of olive variation has been preserved in Iran, which is represented by a few varieties, currently under cultivation in small favorable areas, and a wide set of ecotypes. These ecotypes occur as patchy individual trees or groves, most likely propagated by seeds, in an uncertain state of cultivation that are now abandoned or returned to natural conditions. They are presumably related to the remaining survivors of ancient cultivated olives or trees planted for religious purposes as forms of popular worship. Their occurrence close to settlements where the olive was probably cultivated thousands of years ago, in the western Iranian provinces, may explain the permanence of this important varietal patrimony that human devastations, climatic and historical changes have not completely destroyed but brought to the brink of extinction.

Most Iranian varieties and ecotypes occur in regions representing the cradle of multiple civilizations and at meeting points of ancient eastern and western routes, currently showing extreme pedo-climatic conditions, thus providing a new framework for *Olea europaea* variability distribution. But these trees are endangered because of their fragile environmental and growth conditions, the limited number of plants available for each genotype and the recent massive introduction of alien varieties.

Initiatives for the conservation of this environmental, historical, cultural and natural heritage resource should be mandatory. It is expected that it will soon be possible to perform a deeper exploration in all Iranian provinces for the collection and preservation of the most interesting ecotypes and to start their detailed characterization.

## Supporting Information

Table S1 **List of primers developed to amplify and detect chloroplast SNP and SSR markers.**
(DOCX)

Table S2 **Chloroplast genotyping data based on 24 length (including 29 polymorphisms) and 20 SNP markers.**
(DOC)

Table S3 **Distribution of private (in-range and out-of-range) alleles within the Iranian populations at each locus.**
(DOCX)

Table S4 **Percentage of private alleles and their occurrence in different populations.**
(DOCX)

Table S5 **Results of the uniparental parentage analysis.** Parent and potential offspring identities, typed and compared loci, number of mismatches and LOD score values are reported.
**(**DOCX)
