# Supplementary material for: High Genetic Diversity Detected in Olives beyond the Boundaries of the Mediterranean Sea
Source: PLoS One. 2014 Apr 7;9(4):e93146. doi: 10.1371/journal.pone.0093146 (PMC3977848; doi:10.1371/journal.pone.0093146)
Supplement: Table S1 — List of primers developed to amplify and detect chloroplast SNP and SSR markers. (DOCX) [file pone.0093146.s001.docx]

**Table S1.** List of primers developed to amplify and detect chloroplast SNP and SSR markers.

| **SNP primers** | **Sequence (5' to 3')** |
| --- | --- |
| P1_FW | TTCTTTCTTCGTCTTTACAA |
| P1_REV | TTCGCCCATAACTAAAATGA |
| P2_FW | GTGAGATATTTTTATCTGAGAG |
| P2_REV | CCATCTTTTGCGAAGTTTTT |
| P3_FW | AAATAAAGGAGCAATAACGC |
| P3_REV | GAAAATAAAGGAGCAATAAAC |
| P7_FW | AAAAAGTGTAGATGGGAGAA |
| P7_REV | ATCCAGTTTGTTTATTCCAC |
| P9_FW | ATAACTCGTACAACGGATTA |
| P9_REV | AGAAAGAAAGACTCCTTACA |
| P14_FW | CTGTTATGATTTTAGTGTTTT |
| P14_REV | AGGAGGGTTCATTTTATTT |
| P19_FW | CTTGTCTATGTATTGTTCCA |
| P19_REV | CATGGTTACAGGAGTCTATC |
| P21_FW | GAATGATTGGTCCGTCAGAA |
| P21_REV | TTCCCGTCTTAGTGTGAGAT |
| P23_FW | ATAAATCGCCCCGAAGAGAGT |
| P23_REV | CCATTCGACCATATCCGCAAT |
| P24_FW | GGGCAAGTGTTCGGATCTATT |
| P24_REV | AGCAGCTCTATCTAAGACCTT |
| P26_FW | GACTGATGGGGCCAACAAACA |
| P26_REV | TGAAGATCACGGGGCGTTTCG |
| P27_FW | CGAACCTGAACTCTATTTAC |
| P27_REV | TCGATATGCTGAAATAAAA |
| P29_FW | TCTTGTTGTCGATTATGATTT |
| P29_REV | ATAAATCTACCTATTATGACT |
| P30_FW | GGTGTTCTGAATCAATCAAT |
| P30_REV | TCGTCTAAATGACAAAGTAG |
| P33_FW | CCTCGGGAGCTAATGAAAC |
| P33_REV | ACTAGAATTTTTGGTGCGA |
| P34_FW | CCCATAGGTTTGATCCTGTAG |
| P34_REV | GGGATAGGGATAGAGGAAGAG |
| P35_FW | GCCCACATACGACGAAGATTT |
| P35_REV | TGCGTGGATCATACCTTTTGT |
| P38_FW | ATAGAAGAGCAAATAGAAAA |
| P38_REV | GAAAATCAAAAAGTGAGAAAG |
| P39_FW | CTGGGAATATTAATGATAGA |
| P39_REV | AAAGAAGAAATGCTCGATTA |
| P40_FW | CACTTTTGATAACCCATTTT |
| P40_REV | CCATTTTTAAGGAACTCG |
| **SnapShot primers*** |  |
| P1-SNSH | TCGCCCATAACTAAAATGATATCAT |
| P2-SNSH | GAGAGATAAAAATATTAAGCAAAAATTC |
| P3-SNSH | GGAGCAATAACGCCCTCTTGA |
| P7-SNSH | TAGATGGGAGAATAAGAATTGGAA |
| P9-SNSH | GAGAAAGAAAGACTCCTTACATTAT |
| P14-SNSH | CAGCAAAAGATAAAACTTTAGTTATT |
| P19-SNSH | CATAACCGTCGAGGTGAAGTC |
| P21_SNSH | GTGAGATAGAGATAAGGATATCTC |
| P23-SNSH | GAAGAGAGTCTCTGGCCCGA |
| P24-SNSH | TTCTAATTGAGATATGAATCTACAC |
| P26-SNSH | CGTTTCGAATAAAAGAAGACGCC |
| P27-SNSH | TTAGGATTCATTATTTCTATATCATC |
| P29-SNSH | TTCTACGGAATTCCTTGTACGG |
| P30_SNSH | TTTCGTCTAAATGACAAAGTAGATTT |
| P33-SNSH | AGCTAATGAAACTATTTTAGTAAAATT |
| P34_SNSH | GAACGAAGGGTACGAAATCAATC |
| P35-SNSH | CACATACGACGAAGATTTTTTGTT |
| P38-SNSH | TACTAAAATCATTGAAACTAATTGATT |
| P39-SNSH | CTGGGAATATTAATGATAGATAAAAAT |
| P40-SNSH | AAAATTTCAAAAGAAATATTTTCTACTT |
| **cpSSR primers** | **Sequence (5' to 3')** |
| cpSSR_P4_FW | TCCCCTTTATTTCTTTTTCGGTA |
| cpSSR_P4_REV | CGTGCTAACCTTGGTATGGAA |
| cpSSR_P5_FW | GAGAGAGAGAGAATTGTCAAAATGG |
| cpSSR_P5_REV | TTCCATTGTATTCCTTTCTCAACA |
| cpSSR_P8_FW | GGGTCTCCCACAATATCGAA |
| cpSSR_P8_REV | CAGGATTACGTCCTGGATCAT |
| cpSSR_P10-13_FW | ATCAACGGAACCGGAAAGA |
| cpSSR_P10-13_REV | TTTCTTCTTTTTGTTGTTGCTTGA |
| cpSSR_P15_FW | TTGTTCAATACGTTCACGGATAA |
| cpSSR_P15_REV | GCCTTACAAGGAGCTTTAGGAA |
| cpSSR_P16-17-18_FW | GGTGCCATTTTAGGATTCCA |
| cpSSR_P16-17-18_REV | TTCGTCTATCTACGGCCAATC |
| cpSSR_P20_FW | TTAGACTCGCGGGATCTTTC |
| cpSSR_P20_REV | TCTGACGGACCAATCATTCC |
| cpSSR_P22_FW | CCTATTCGTCCCAGATCCAA |
| cpSSR_P22_REV | AAGAAGTTTGCAAGAAGTTTGACC |
| cpSSR_P25_FW | CGCGAGCAGCCATTACTAAG |
| cpSSR_P25_REV | CTTTGGGAAGAGCCAATCAA |
| cpSSR_P28_FW | CAGCATATCGATTTACGCTTACG |
| cpSSR_P28_REV | CCAAATACATCATTATTGTATACTC |
| cpSSR_P31_FW | CGATGCGAACCACTCTTTTT |
| cpSSR_P31_REV | GATTGTGTCTCACGCATATACCTT |
| cpSSR_P32_FW | AAGGTATATGCGTGAGACACAATC |
| cpSSR_P32_REV | TTTCATTAGCTCCCGAGGTATTAT |
| cpSSR_P36_FW | CAAGAAGTTACAATTGGTCAAATCA |
| cpSSR_P36_REV | TTCAAAATGAATCCGTAGTTTCC |
| cpSSR_P37_FW | AAATTGACTCATTTCGAAAATCAAA |
| cpSSR_P37_REV | AAATAAGACTCCCCGCCTTG |

*Specific primers for single base amplification by Snap Shot analysis.
