# Supplementary material for: High Genetic Diversity Detected in Olives beyond the Boundaries of the Mediterranean Sea
Source: PLoS One. 2014 Apr 7;9(4):e93146. doi: 10.1371/journal.pone.0093146 (PMC3977848; doi:10.1371/journal.pone.0093146)
Supplement: Table S2 — Chloroplast genotyping data based on 24 length (including 29 polymorphisms) and 20 SNP markers. (DOC) [file pone.0093146.s002.doc]

**Table S2.** Chloroplast genotyping data based on 24 length (including 29 polymorphisms) and 20 SNP markers.

|  | **Length chloroplast polymorphisms*** | | | | | | | | | | | | | | | | | | | | | | | |
| --- | --- | --- | --- | --- | --- | --- | --- | --- | --- | --- | --- | --- | --- | --- | --- | --- | --- | --- | --- | --- | --- | --- | --- | --- |
|  | **P4** | **P5** | **P8** | **P10-11-12-13** | **P15** | **P16-17-18** | **P20** | **P22** | **P25** | **P28** | **P31** | **P32** | **P36** | **P37** | **6** | **21** | **31** | **38** | **41** | **42** | **46** | **47** | **54** | **58** |
| **Haplotype E.1.1** | 287 | 217 | 298 | 456 | 239 | 217 | 232 | 148 | 244 | 233 | 205 | 130 | 268 | 239 | 173 | 103 | 132 | 109 | 171 | 138 | 112 | 155 | 238 | 235 |
| Iranian ecotypes | 287 | 217 | 298 | 456 | 239 | 217 | 232 | 148 | 244 | 233 | 205 | 130 | 268 | 239 | 173 | 103 | 132 | 109 | 171 | 138 | 112 | 155 | 238 | 235 |
| Iranian main cultivars | 287 | 217 | 298 | 456 | 239 | 217 | 232 | 148 | 244 | 233 | 205 | 130 | 268 | 239 | 173 | 103 | 132 | 109 | 171 | 138 | 112 | 155 | 238 | 235 |
| **Haplotype *Olea cuspidata* India** | 287 | 216 | 297 | 464 | 240 | 217 | 231 | 145 | 245 | 232 | 199 | 116 | 268 | 238 | 173 | 102 | 132 | 110 | 170 | 138 | 111 | 156 | 238 | 236 |
| *Hooshak*_Sistan-Baloochestan | 287 | 216 | 297 | 464 | 240 | 217 | 231 | 145 | 245 | 232 | 199 | 116 | 268 | 238 | 173 | 102 | 132 | 110 | 170 | 138 | 111 | 156 | 238 | 236 |
| *Aghin*_Kerman | 287 | 216 | 297 | 464 | 240 | 217 | 231 | 145 | 245 | 232 | 199 | 116 | 268 | 238 | 173 | 102 | 132 | 110 | 170 | 138 | 111 | 156 | 238 | 236 |
| **Haplotype *Olea cuspidata* Nepal** | 287 | 216 | 297 | 464 | 241 | 217 | 231 | 145 | 245 | 232 | 199 | 130 | 268 | 238 | 173 | 102 | 132 | 109 | 170 | 138 | 112 | 156 | 238 | 235 |
| *Bokhoon-1*_Hormozgan | 287 | 216 | 297 | 464 | 241 | 217 | 231 | 145 | 245 | 232 | 199 | 130 | 268 | 238 | 173 | 102 | 132 | 109 | 170 | 138 | 112 | 156 | 238 | 235 |
| *Bokhoon-2*_Hormozgan | 287 | 216 | 297 | 464 | 241 | 217 | 231 | 145 | 245 | 232 | 199 | 130 | 268 | 238 | 173 | 102 | 132 | 109 | 170 | 138 | 112 | 156 | 238 | 235 |
| *Bokhoon-3*_Hormozgan | 287 | 216 | 297 | 464 | 241 | 217 | 231 | 145 | 245 | 232 | 199 | 130 | 268 | 238 | 173 | 102 | 132 | 109 | 170 | 138 | 112 | 156 | 238 | 235 |
| *Bokhoon-4*_Hormozgan | 287 | 216 | 297 | 464 | 241 | 217 | 231 | 145 | 245 | 232 | 199 | 130 | 268 | 238 | 173 | 102 | 132 | 109 | 170 | 138 | 112 | 156 | 238 | 235 |
| *Bokhoon-5*_Hormozgan | 287 | 216 | 297 | 464 | 241 | 217 | 231 | 145 | 245 | 232 | 199 | 130 | 268 | 238 | 173 | 102 | 132 | 109 | 170 | 138 | 112 | 156 | 238 | 235 |
| *Bokhoon-6*_Hormozgan | 287 | 216 | 297 | 464 | 241 | 217 | 231 | 145 | 245 | 232 | 199 | 130 | 268 | 238 | 173 | 102 | 132 | 109 | 170 | 138 | 112 | 156 | 238 | 235 |
| *Bokhoon-7*_Hormozgan | 287 | 216 | 297 | 464 | 241 | 217 | 231 | 145 | 245 | 232 | 199 | 130 | 268 | 238 | 173 | 102 | 132 | 109 | 170 | 138 | 112 | 156 | 238 | 235 |
| *Bokhoon-8*_Hormozgan | 287 | 216 | 297 | 464 | 241 | 217 | 231 | 145 | 245 | 232 | 199 | 130 | 268 | 238 | 173 | 102 | 132 | 109 | 170 | 138 | 112 | 156 | 238 | 235 |
| *Bokhoon-9*_Hormozgan | 287 | 216 | 297 | 464 | 241 | 217 | 231 | 145 | 245 | 232 | 199 | 130 | 268 | 238 | 173 | 102 | 132 | 109 | 170 | 138 | 112 | 156 | 238 | 235 |
| *Bokhoon-10*_Hormozgan | 287 | 216 | 297 | 464 | 241 | 217 | 231 | 145 | 245 | 232 | 199 | 130 | 268 | 238 | 173 | 102 | 132 | 109 | 170 | 138 | 112 | 156 | 238 | 235 |
| *Beerk-I*_Sistan-Baloochestan | 287 | 216 | 297 | 464 | 241 | 217 | 231 | 145 | 245 | 232 | 199 | 130 | 268 | 238 | 173 | 102 | 132 | 109 | 170 | 138 | 112 | 156 | 238 | 235 |
| *Beerk-II*_Sistan-Baloochestan | 287 | 216 | 297 | 464 | 241 | 217 | 231 | 145 | 245 | 232 | 199 | 130 | 268 | 238 | 173 | 102 | 132 | 109 | 170 | 138 | 112 | 156 | 238 | 235 |
| *Torang-121*_Kerman | 287 | 216 | 297 | 464 | 241 | 217 | 231 | 145 | 245 | 232 | 199 | 130 | 268 | 238 | 173 | 102 | 132 | 109 | 170 | 138 | 112 | 156 | 238 | 235 |
| *Torang-122*_Kerman | 287 | 216 | 297 | 464 | 241 | 217 | 231 | 145 | 245 | 232 | 199 | 130 | 268 | 238 | 173 | 102 | 132 | 109 | 170 | 138 | 112 | 156 | 238 | 235 |
| *Torang-123*_Kerman | 287 | 216 | 297 | 464 | 241 | 217 | 231 | 145 | 245 | 232 | 199 | 130 | 268 | 238 | 173 | 102 | 132 | 109 | 170 | 138 | 112 | 156 | 238 | 235 |
| *Torang-IV*_Kerman | 287 | 216 | 297 | 464 | 241 | 217 | 231 | 145 | 245 | 232 | 199 | 130 | 268 | 238 | 173 | 102 | 132 | 109 | 170 | 138 | 112 | 156 | 238 | 235 |
| *Torang-128*_Kerman | 287 | 216 | 297 | 464 | 241 | 217 | 231 | 145 | 245 | 232 | 199 | 130 | 268 | 238 | 173 | 102 | 132 | 109 | 170 | 138 | 112 | 156 | 238 | 235 |
|  | **Chloroplast SNPs** | | | | | | | | | | | | | | | | | | | | | | | |
|  | **P1** | **P2** | **P3** | **P7** | **P9** | **P14** | **P19** | **P21** | **P23** | **P24** | **P26** | **P27** | **P29** | **P30** | **P33** | **P34** | **P35** | **P38** | **P39** | **P40** |  |  |  |  |
| **Haplotype E.1.1** | G | T | T | A | T | T | C | C | C | A | C | A | C | T | T | T | A | C | A | G |  |  |  |  |
| Iranian ecotypes | G | T | T | A | T | T | C | C | C | A | C | A | C | T | T | T | A | C | A | G |  |  |  |  |
| Iranian main cultivars | G | T | T | A | T | T | C | C | C | A | C | A | C | T | T | T | A | C | A | G |  |  |  |  |
| **Haplotype *Olea cuspidata* India** | A | A | T | A | C | T | C | T | C | A | C | G | C | G | T | A | G | C | A | G |  |  |  |  |
| *Hooshak*_Sistan-Baloochestan | A | A | T | A | C | T | C | T | C | A | C | G | C | G | T | A | G | C | A | G |  |  |  |  |
| *Aghin*_Kerman | A | A | T | A | C | T | C | T | C | A | C | G | C | G | T | A | G | C | A | G |  |  |  |  |
| **Haplotype *Olea cuspidata* Nepal** | A | T | T | A | C | T | C | T | C | A | C | G | C | G | T | T | G | C | A | G |  |  |  |  |
| *Bokhoon-1*_Hormozgan | A | T | T | A | C | T | C | T | C | A | C | G | C | G | T | T | G | C | A | G |  |  |  |  |
| *Bokhoon-2*_Hormozgan | A | T | T | A | C | T | C | T | C | A | C | G | C | G | T | T | G | C | A | G |  |  |  |  |
| *Bokhoon-3*_Hormozgan | A | T | T | A | C | T | C | T | C | A | C | G | C | G | T | T | G | C | A | G |  |  |  |  |
| *Bokhoon-4*_Hormozgan | A | T | T | A | C | T | C | T | C | A | C | G | C | G | T | T | G | C | A | G |  |  |  |  |
| *Bokhoon-5*_Hormozgan | A | T | T | A | C | T | C | T | C | A | C | G | C | G | T | T | G | C | A | G |  |  |  |  |
| *Bokhoon-6*_Hormozgan | A | T | T | A | C | T | C | T | C | A | C | G | C | G | T | T | G | C | A | G |  |  |  |  |
| *Bokhoon-7*_Hormozgan | A | T | T | A | C | T | C | T | C | A | C | G | C | G | T | T | G | C | A | G |  |  |  |  |
| *Bokhoon-8*_Hormozgan | A | T | T | A | C | T | C | T | C | A | C | G | C | G | T | T | G | C | A | G |  |  |  |  |
| *Bokhoon-9*_Hormozgan | A | T | T | A | C | T | C | T | C | A | C | G | C | G | T | T | G | C | A | G |  |  |  |  |
| *Bokhoon-10*_Hormozgan | A | T | T | A | C | T | C | T | C | A | C | G | C | G | T | T | G | C | A | G |  |  |  |  |
| *Beerk-I*_Sistan-Baloochestan | A | T | T | A | C | T | C | T | C | A | C | G | C | G | T | T | G | C | A | G |  |  |  |  |
| *Beerk-II*_Sistan-Baloochestan | A | T | T | A | C | T | C | T | C | A | C | G | C | G | T | T | G | C | A | G |  |  |  |  |
| *Torang-121*_Kerman | A | T | T | A | C | T | C | T | C | A | C | G | C | G | T | T | G | C | A | G |  |  |  |  |
| *Torang-122*_Kerman | A | T | T | A | C | T | C | T | C | A | C | G | C | G | T | T | G | C | A | G |  |  |  |  |
| *Torang-123*_Kerman | A | T | T | A | C | T | C | T | C | A | C | G | C | G | T | T | G | C | A | G |  |  |  |  |
| *Torang-IV*_Kerman | A | T | T | A | C | T | C | T | C | A | C | G | C | G | T | T | G | C | A | G |  |  |  |  |
| *Torang-128*_Kerman | A | T | T | A | C | T | C | T | C | A | C | G | C | G | T | T | G | C | A | G |  |  |  |  |

* Chloroplast polymorphisms with **P** are related to Mariotti et al. 2010; chloroplast polymorphisms with just the numbers are coming from Besnard et al. 2011.
