# Supplementary material for: High Genetic Diversity Detected in Olives beyond the Boundaries of the Mediterranean Sea
Source: PLoS One. 2014 Apr 7;9(4):e93146. doi: 10.1371/journal.pone.0093146 (PMC3977848; doi:10.1371/journal.pone.0093146)
Supplement: Table S3 — Distribution of private (in-range and out-of-range) alleles within the Iranian populations at each locus. (DOCX) [file pone.0093146.s003.docx]

**Table S3**. Distribution of private (in-range and out-of-range) alleles within the Iranian populations at each locus.

| **Loci** | **Main cultivars** | **Ecotypes** | **Ecotypes + Main Cultivars** | **Total Iran**^(1)^ | ***cuspidata* shared with ecotypes and main cultivars** | ***cuspidata*** |
| --- | --- | --- | --- | --- | --- | --- |
| **DCA3** | 0-0^(2)^ | 1-7 | 2-0 | 4-14 | 0-3 | 1-4 |
| **DCA5** | 0-0 | 0-1 | 0-1 | 0-2 | 0-0 | 0-0 |
| **DCA9** | 0-0 | 0-0 | 2-0 | 7-2 | 3-0 | 2-2 |
| **DCA14** | 0-0 | 0-0 | 1-0 | 1-4 | 0-1 | 0-3 |
| **DCA16** | 0-0 | 3-5 | 2-1 | 14-6 | 2-0 | 7-0 |
| **DCA18** | 0-0 | 0-2 | 0-0 | 1-4 | 0-0 | 1-2 |
| **EMO-90** | 0-0 | 0-0 | 1-0 | 3-1 | 2-1 | 0-0 |
| **GAPU71B** | 0-0 | 0-0 | 0-0 | 4-1 | 1-0 | 3-1 |
| **GAPU101** | 1-0 | 0-0 | 0-1 | 5-2 | 2-0 | 2-1 |
| **GAPU103A** | 1-0 | 0-0 | 2-0 | 5-0 | 1-0 | 1-0 |
| **UDO-043** | 0-0 | 0-2 | 0-0 | 0-3 | 0-0 | 0-1 |
| **Total** | 2-0 | 4-17 | 10-3 | 44-39=83 | 11-5=16 | 17-14=31 |

^(1)^Numbers refer to private alleles to one, two or all Iranian populations.

^(2)^Number of private alleles to each Iranian population (in-range - out-of-range).
