# Supplementary material for: High Genetic Diversity Detected in Olives beyond the Boundaries of the Mediterranean Sea
Source: PLoS One. 2014 Apr 7;9(4):e93146. doi: 10.1371/journal.pone.0093146 (PMC3977848; doi:10.1371/journal.pone.0093146)
Supplement: Table S4 — Percentage of private alleles and their occurrence in different populations. (DOCX) [file pone.0093146.s004.docx]

**Table S4**. Percentage of private alleles and their occurrence in different populations.

| **Private alleles for each population or group** | **Percentage of private alleles**^(1)^ | **Percentage of occurrence**^(2)^ |
| --- | --- | --- |
| Ecotypes | 15,33% | 2.08% |
| Main cultivars | 2,50% | 0.27% |
| Ecotypes and main cultivars | 8,97% | 12.23% |
| TOTAL | 24,83% | 14.58% |
| Ecotypes and *cuspidata* | 4,52% | 1.55% |
| Cultivars and *cuspidata* | 1,43% | 0.07% |
| Ecotypes, cultivars and *cuspidata* | 3,24% | 5.58% |
| TOTAL | 8,65% | 7.19% |
| **Alleles shared by Iranian samples**  **and Mediterranean cultivars** | | |
| Iranian cultivars and ecotypes vs. Mediterranean cultivars | 50,27% | 78.23% |
| *cuspidata* vs. Mediterranean cultivars | 24,73% | 57.51% |

^(1)^ Percentages refer to the total number of alleles per locus of each population or group.

^(2)^ Percentages refer to the total number of alleles of each population or group.
