# Supplementary material for: High Genetic Diversity Detected in Olives beyond the Boundaries of the Mediterranean Sea
Source: PLoS One. 2014 Apr 7;9(4):e93146. doi: 10.1371/journal.pone.0093146 (PMC3977848; doi:10.1371/journal.pone.0093146)
Supplement: Table S5 — Results of the uniparental parentage analysis. Parent and potential offspring identities, typed and compared loci, number of mismatches and LOD score values are reported. (DOCX) [file pone.0093146.s005.docx]

**Table S5**. Results of the uniparental parentage analysis. Parent and potential offspring identities, typed and compared loci, number of mismatches and LOD score values are reported.

| **Parent** | **Offspring** | **Loci typed** | **Pair loci compared** | **Pair loci mismatching** | **Pair LOD score** |
| --- | --- | --- | --- | --- | --- |
| FISHOMI | SHENGEH-III | 10 | 9 | 0 | 9,52E+00 |
| FISHOMI | KHORMA-II | 10 | 10 | 1 | 4,13E+00 |
| FISHOMI | ROWGHANI-III | 10 | 10 | 1 | 3,40E+00 |
| FISHOMI | ROWGHANI-I | 10 | 10 | 2 | 1,18E-01 |
| FISHOMI | KHORMA-IV | 10 | 10 | 2 | 1,87E-01 |
| FISHOMI | Park-e-Sarpol-1_Kermanshah | 10 | 9 | 0 | 1,09E+01 |
| FISHOMI | Gilan-e-Gharb-1_Kermanshah | 10 | 9 | 1 | 2,07E+00 |
| FISHOMI | Dezful-Safiabad_Khuzestan | 10 | 9 | 1 | 2,71E+00 |
| FISHOMI | Dastjerd-4_Zanjan | 10 | 9 | 1 | 4,01E+00 |
| GOLOOLEH-I | ROWGHANI-I | 10 | 10 | 1 | 1,75E+00 |
| GOLOOLEH-I | Banavare-3_Kermanshah | 10 | 10 | 1 | 1,15E+00 |
| GOLOOLEH-I | Banavare-5_Kermanshah | 10 | 9 | 1 | 2,63E+00 |
| GOLOOLEH-I | Banavare-6_Kermanshah | 10 | 10 | 1 | 6,05E+00 |
| GOLOOLEH-I | Dastjerd-4_Zanjan | 10 | 9 | 1 | 4,07E+00 |
| ROWGHANI-I | KHORMA-II | 11 | 11 | 0 | 8,36E+00 |
| ROWGHANI-I | GOLOOLEH-I | 11 | 10 | 1 | 1,75E+00 |
| ROWGHANI-I | GOLOOLEH-II | 11 | 11 | 1 | 3,68E+00 |
| ROWGHANI-I | ROWGHANI-III | 11 | 11 | 1 | 7,57E+00 |
| ROWGHANI-I | SHENGEH-III | 11 | 10 | 1 | 3,71E+00 |
| ROWGHANI-I | Dastjerd-4_Zanjan | 11 | 10 | 0 | 1,18E+01 |
| ROWGHANI-I | Banavare-7_Kermanshah | 11 | 11 | 2 | 9,83E-01 |
| Dastjerd-4_Zanjan | ROWGHANI-I | 10 | 10 | 0 | 1,18E+01 |
| Dastjerd-4_Zanjan | SHENGEH-III | 10 | 10 | 0 | 1,08E+01 |
| Dastjerd-4_Zanjan | FISHOMI | 10 | 9 | 1 | 4,01E+00 |
| Dastjerd-4_Zanjan | GOLOOLEH-I | 10 | 9 | 1 | 4,07E+00 |
| Dastjerd-4_Zanjan | KHORMA-II | 10 | 10 | 1 | 3,16E+00 |
| Dastjerd-4_Zanjan | KHORMA-IV | 10 | 10 | 1 | 4,08E+00 |
| Dastjerd-4_Zanjan | Park-e-Sarpol-1_Kermanshah | 10 | 9 | 0 | 9,91E+00 |
| Dastjerd-4_Zanjan | Dezful-Safiabad_Khuzestan | 10 | 9 | 1 | 2,36E+00 |
